# The Role of Cardiokines in Heart Diseases: Beneficial or Detrimental?

**DOI:** 10.1155/2018/8207058

**Published:** 2018-03-18

**Authors:** Ye-Shun Wu, Bin Zhu, Ai-Lin Luo, Ling Yang, Chun Yang

**Affiliations:** ^1^Department of Cardiology, The Third Affiliated Hospital of Soochow University, Changzhou 213003, China; ^2^Department of Critical Care Medicine, The Third Affiliated Hospital of Soochow University, Changzhou 213003, China; ^3^Department of Anesthesiology, Tongji Hospital, Tongji Medical College, Huazhong University of Science & Technology, Wuhan 430030, China

## Abstract

Cardiovascular disease remains the leading cause of morbidity and mortality, imposing a major disease burden worldwide. Therefore, there is an urgent need to identify new therapeutic targets. Recently, the concept that the heart acts as a secretory organ has attracted increasing attention. Proteins secreted by the heart are called cardiokines, and they play a critical physiological role in maintaining heart homeostasis or responding to myocardial damage and thereby influence the development of heart diseases. Given the critical role of cardiokines in heart disease, they might represent a promising therapeutic target. This review will focus on several cardiokines and discuss their roles in the pathogenesis of heart diseases and as potential therapeutics.

## 1. Introduction

Despite an obvious decrease in the number of deaths attributable to cardiovascular disease (CVD) during the preceding decades, it remains the primary killer worldwide and, unfortunately, the hospitalization rate in patients less than 55 years old has not been improved [1]. With changing lifestyles and an aging population, cardiovascular risk factors have become more prevalent, and the number of people living with CVD is increasing, thereby causing a seemingly unbearable economic burden for society [2]. It is therefore necessary to identify new strategies to achieve more accurate diagnosis, which could result in better treatments for CVD.

Cardiac myocytes have been reported to synthesize and release natriuretic peptides [3–5]. In addition to its role as a mechanically beating organ, the role of the heart as a secretory organ has attracted increasing attention. It has been well recognized that investigating the pathogenesis of heart failure (HF) has transformed from an investigation of cardiac hemodynamics to neuroendocrinological assessments. Cardiac dysfunction can significantly activate the natriuretic peptide system [4], and atrial natriuretic peptide (ANP) and brain natriuretic peptide (BNP) are both closely related to the motion of the cardiac wall (such as under conditions of excessive blood, assumption of the trendelenburg position, or increased central venous pressure) and improving the signal transduction between the heart and peripheral organs. Given that this paracrine/autocrine signaling within the heart plays a critical physiological role in the process of cardiac diseases, there is an urgent need to identify novel therapeutic targets based on the secretory function of the heart.

A growing body of evidence showed that the peptides or proteins secreted from cardiac cells can be considered cardiokines [6]. Most cardiokines, as important mediators, play pivotal roles in maintaining healthy heart homeostasis or in the response to myocardial damage. It has been reported that cardiokines not only have physiological involvement in the stress response, damage repair, and myocardial remodeling, but could also participate in protein synthesis in distal organ tissues and systemic metabolic processes [7, 8]. Additionally, cardiokines are differentially expressed in various physiological conditions of the heart, and these secreted cardiokines are intended to maintain healthy cardiac function through paracrine/autocrine pathways or affect the response of cardiomyocytes and cardiac fibroblasts (CFs) to pathological abnormalities caused by heart damage or other associated inflammatory processes, ultimately eliciting a protective or harmful effect on cardiac function [7, 8].

Many researchers have realized that cardiokines could act as biomarkers to evaluate cardiac function, and therefore contribute to clinical diagnosis, and provide novel therapeutic targets for cardiac diseases. Increasing attention has been paid by researchers in this field to identifying novel cardiokines, with a view to understanding abnormalities in intercellular communication to better diagnose heart disease. In addition to regular laboratory examinations, advanced techniques including gene expression analysis, array screening, cloning, and other methods provide advanced approaches to identify novel cardiokines and determine the networks between cardiokines that are dysregulated during cardiac stress [7, 8]. In this review, we briefly introduce several cardiokines and discuss their roles in the pathogenesis and treatment of cardiac diseases. Furthermore, we summarize the physiological effects of these cardiokines in cardiac diseases in Table 1.

## 2. The Beneficial Role of Cardiokines in CVD

### 2.1. Natriuretic Peptides

Natriuretic peptides, and in particular ANP and BNP, secreted by the cardiovascular system, have a particularly large impact on the occurrence and development of CVD in a paracrine/autocrine manner [9, 10]. It is well recognized that ANP and BNP are useful for the clinical diagnosis, treatment, and prognosis of CVD [9, 10]. There is evidence that ANP is significantly elevated in patients with left ventricular dysfunction which is independent of clinical symptoms, and that the ANP levels in the circulation are negatively correlated with ejection fraction (EF) [11]. Interestingly, increased levels of ANP in the circulation are positively correlated with the severity of congestive heart failure (CHF), whereas ANP levels are significantly decreased after an improvement in CHF symptoms.

BNP, also known as B-type natriuretic peptide, is mainly secreted by ventricular myocytes [12]. Although BNP has a variety of biological actions, cardiomyocytes only directly synthesize the precursor of BNP (the 108 amino acid proBNP) [12, 13]. ProBNP, which is initially stored in cardiomyocytes, is released and instantly decomposes into BNP and inactive NT-proBNP in equimolar quantities when the ventricular walls experience stretching forces or ventricular pressure is increased [3]. It therefore appears that BNP and its precursor play a clinically significant role in response to various CVDs such as HF, hypertension, and arrhythmias [14, 15]. In addition, BNP contributes to better diagnosis of acute HF, and in particular HF classification [16]. Similarly, BNP is closely associated with the prognosis of chronic HF and also could be used as an independent prognostic marker for CVD. The European Society of Cardiology has recommended BNP as an indicator for the diagnosis of HF in 2001, and the 2005 American guidelines for HF further reinforced this recommendation [17]. Theoretically, BNP and NT-proBNP are equally significant for CVD diagnosis. A recent systematic review suggested that BNP strongly correlates with NT-proBNP, and joint measurements could improve the accuracy and reliability of the diagnosis of acute or chronic HF [18]. Compared with BNP, NT-proBNP possesses a longer half-life and is more stable in plasma. NT-proBNP levels are closely related to newly synthesized rather than stored BNP and NT-proBNP preferentially reflects the activation of the BNP pathway [19].

### 2.2. Interleukin-33

The inflammatory response is thought to be one of the most important mechanisms in the process of atherosclerosis. Abnormalities in the levels of various inflammatory cytokines have been found in patients with acute coronary syndrome [20, 21]. Extremely elevated interleukin (IL) levels in the heart and myocardial necrosis during acute myocardial infarction (MI) indicate that ILs act as an important regulatory factor in acute MI [22].

IL-33, mainly secreted by CFs, is a paracrine signaling molecule involved in crosstalk between fibroblasts and cardiomyocytes, and it is also the specific ligand for soluble ST2 (sST2), which is confirmed to be a cardiomyocyte protein. Mechanical traction and stimulation remarkably upregulate the expression of IL-33 in cardiomyocytes and fibroblasts, as well as levels of ST2 (sST2 levels are significantly higher than those of ST2), and then sST2 exhibits competitive inhibition, thereby blocking the IL-33/ST2 signaling pathway and attenuating the protective effect of IL-33 on cardiomyocyte hypertrophy and myocardial fibrosis [23, 24]. Furthermore, an aldosterone receptor antagonist indirectly upregulates IL-33 expression by reducing ST2 levels, enhancing the IL-33/ST2 signaling pathway and then reducing inflammation and fibrosis after MI [25]. In addition, serum ST2 levels are closely associated with the prognosis of MI and HF [26], and they have been recommended as a biomarker for additional risk stratification in the American Heart Failure Guidelines 2013 [27]. Studies of coronary heart disease patients also show that genetic polymorphisms of these inflammatory cardiokines could increase the risk of coronary heart disease [28, 29].

### 2.3. Follistatin

Follistatin is an extracellular modulator that selectively binds to proteins of the transforming growth factor-*β* super family (TGF-*β*; discussed later). Follistatin-like 1 (FSTL1), also known as transforming growth factor *β*-stimulated clone 36 (TSC-36) [30], has been identified as a cardioprotective factor that could protect cardiomyocytes and decrease apoptosis induced by ischemia/reperfusion (IR) injury [31, 32]. The physiological mechanisms underlying FSTL1 action are quite different from those of other follistatins. A recent study revealed that the expression of FSTL1 in the ischemic area after MI is obviously increased in fibroblasts, but not in cardiomyocytes [33]. Compared with wild-type mice, the activation and differentiation of myofibroblasts in FSTL1 gene-knockout mice were attenuated, and the accelerated formation of extracellular matrix (ECM), such as collagen and fibrous proteins, in the ischemic heart was obviously reduced, suggesting an increased risk of mortality with cardiac rupture. These findings indicate that FSTL1 could stimulate the activation of fibroblasts and protect against cardiac rupture and left ventricular remodeling [34]. Interestingly, Altekoester and colleagues reported that bioengineered FSTL1 patches reduce heart scarring and induce angiogenesis, which may provide an effective strategy for reducing the risk of cardiac rupture and unfavorable remodeling after MI [35].

In a study by Tanaka et al. [36, 37], FSTL1 expression induced by cardiac stress was described to modulate cardiac hypertrophy, while FSTL1 knockout mice showed more serious cardiac hypertrophy and cardiac dysfunction after HF. Similarly, Ogura and coworkers [38] demonstrated that recombinant FSTL1 administered in mice or pig models could remarkably reduce the proportion of the MI region after IR, subsequently inhibiting apoptosis and the inflammatory response via adenosine 5′-monophosphate- (AMP-) activated protein kinase and bone morphogenetic protein-4-dependent mechanisms. Moreover, overexpression of FSTL1 also minimized the deleterious effects of IR injury [38]. All these findings indicate that FSTL1 may become a therapeutic target for cardiac hypertrophy or other heart diseases.

### 2.4. Fibroblast Growth Factor 21

Fibroblast growth factors (FGFs) play a definite role in inducing angiogenesis, repairing impaired endothelial cells, and promoting vascular smooth muscle cell proliferation [39, 40]. To date, there are 22 identified human or murine FGFs. FGF sequences between different animals have a high relative homology. FGFs transmit signals inside cells through their related external receptors on the cell membrane [41].

FGF21, a new member of the FGF family, consists of 209 amino acids. It is physiologically decomposed into 181 amino acids for maturity. FGF21 is mainly produced and released from the liver, but a recent study indicates that cardiomyocytes and cardiac microvascular endothelial cells (CMECs) might also express FGF21 to improve cardiac remodeling and reduce heart damage [42]. FGF21 specifically binds to FGFR1c in the heart to exert biological effects through activation of its coreceptor *β*-klotho [41]. The N-terminus of FGF21 binds to FGFR1c while the C-terminus binds to *β*-Klotho with a high affinity to form a complex that phosphorylates FGFR1c and activates downstream signal transduction (extracellular regulated protein kinases (ERK) signaling pathway), which explains the intracellular effects of FGF21 [43, 44]. In addition, FGF21 is secreted into the systemic circulation by damaged myocardial or endothelial cells and affects cell surface receptors to regulate lipid metabolism and protect against oxidative stress or inflammatory injury, thereby leading to amelioration of atherosclerosis and protection against ischemic myocardium and IR injury [45].

### 2.5. Secreted Frizzled-Related Protein

Secreted frizzled-related protein-3 (Sfrp-3) is the strongest Wnt signal antagonist in the Sfrps family. Recent studies revealed that Wnt signaling is involved in cardiac hypertrophy [46, 47], as well as in the progression of late atherosclerosis and it is significantly associated with vascular inflammation, endothelial dysfunction, calcification, and intimal thickening [48]. Askevold et al. [49] demonstrated that Sfrp3 levels are markedly elevated in patients with HF, which is consistent with the New York Heart Association (NYHA) functional classification. In contrast, Sfrp3 is significantly decreased in end-stage HF patients after treatment [50]. It is therefore likely that Wnt/Sfrp3 might play a significant role in left ventricular remodeling. Furthermore, mRNA levels of Sfrp3 are elevated in cardiomyocytes, endothelial cells, and CFs in the MI region in mouse models [49, 50]. Sfrp3 appears to be a biomarker reflecting the pathogenesis of HF and a potential therapeutic target for heart diseases.

Similar to Sfrp3, Sfrp2 plays a critical role in inhibiting apoptosis and inflammatory reactions through interfering with Wnt signaling [51]. The expression of Sfrp2 in the murine heart after MI is significantly upregulated, suggesting that Sfrp2 is a stress-inducible cardiokine [52]. Furthermore, an exogenous injection of Sfrp2 at a therapeutic dose could deactivate the activity of bone morphogenic protein 1 (Bmp1)/Tolloid-like metalloproteinase [53], thereby inhibiting collagen deposition at the late stage of MI and improving left ventricular function. Therefore, Sfrp2 is a potential therapeutic target for antifibrosis therapies [54].

### 2.6. Macrophage Migration Inhibitory Factor

Macrophage migration inhibitory factor (MIF) is a highly conserved factor closely related to the inflammatory response. MIF is released from necrotic cardiomyocytes after MI, and the levels in circulation are rapidly increased after stimulation since the cells produce and store MIF before the inflammatory response [55, 56]. Furthermore, increased MIF levels are closely associated with the infarct region [57], making it a potential biomarker. A previous study reported that the −173G/C polymorphism of MIF is associated with coronary heart diseases [58].

After MI, MIFs of different cellular sources play opposing physiological roles. In a study by White et al. [59], mice with MIF deficiency in bone marrow derived-cells had a lower incidence of cardiac rupture after MI, whereas MIF deficiency in somatic/cardiac cells accelerated ventricular dilatation and dysfunction. In conclusion, the majority of MIFs in the infarcted myocardium are from infiltrating inflammatory cells, rather than cardiogenic cells. Inhibiting MIF from inflammatory cells could protect cardiac function and improve MI prognosis. Moreover, MIF protects the heart from short-term hypoxia, but, with the prolongation of ischemia and hypoxia, the protective effect of MIF in the heart is gradually weakened. Meanwhile, the proinflammatory effect of MIF gradually emerges, eventually exacerbating myocardial injury [55]. Collectively, as mentioned previously, this bidirectional effect of MIF may be associated with its different origins.

### 2.7. Neuregulin

Neuregulin (NRG) is a member of the epidermal growth factor (EGF) family and is mainly secreted by microvascular endothelial cells and endocardium in the heart. NRG can promote angiogenesis, reverse myocardial remodeling, and improve apoptosis and oxidative stress. It has recently been reported that NRG is also an important signaling protein in the cardiovascular system and in regulating cardiac development and cardiac function, since the tyrosine kinase receptor of NRG (ErbB) has been detected on the surface of cardiomyocytes [60]. Hedhli et al. demonstrated that hypoxia-reoxygenation could induce myocardial endothelial cells to express and release NRG, and that NRG could protect adult mouse cardiomyocytes against apoptosis during hypoxia-reoxygenation [61]. In addition, NRG may directly improve fibrosis [62] and induce the production and secretion of IL-1*α* and repair factors (like crypto-1), which affect cardiac healing through paracrine signaling [60]. These findings indicate that endothelium-derived NRG has a protective effect in the ischemic myocardium and it may represent a new therapeutic target for heart diseases.

### 2.8. Adrenomedullin

Adrenomedullin (ADM) is a product of vascular endothelial cells, smooth muscle cells, and cardiomyocytes and is thought to be a local factor in controlling vascular tension, cardiac contractility, and renal sodium excretion [63]. Cheung et al. suggested a significant increase of plasma ADM levels in patients with CHF because of neuroendocrine reactions [64].

ADM levels are associated with endothelial injury and can indicate the severity of atherosclerotic endothelial cell injury [65]. In addition, a previous study showed that ADM is beneficial for HF and MI and that short-term treatment using ADM reduces the area of MI and IR injuries because of its antioxidant and antiapoptosis effects [66]. A follow-up study by Nishida et al. demonstrated that a high risk for CVD was associated with abnormal plasma levels of ADM in 121 patients [67]. This study suggested that plasma ADM is an independent predictor of cardiovascular events in high risk patients [68, 69]. In conclusion, ADM is a predicative biomarker for the onset of CVD, and in particular HF.

### 2.9. Protease Inhibitor 16

Protease inhibitor 16 (PI16) is a protein secreted by cardiomyocytes and it might elicit inhibitory effects on myocardial hypertrophy. It is strongly upregulated in the early phase of HF and restrains the growth of cardiomyocytes in vitro and in vivo. Overexpression of PI16 inhibits hypertrophy in cultured cardiomyocytes. In contrast, PI16 knockout activates hypertrophy [70]. Interestingly, it has been determined that the Kruppel-like factor 2- (KLF2-) mediated ERK5-dependent signaling pathway may be involved in PI16 inhibition of endothelial migration and proliferation, which contributes to the maintenance of vascular homeostasis [71]. Adenovirus-mediated PI16 overexpression inhibits the secretion of matrix metalloproteinases (MMPs; discussed later). In addition, inflammatory cytokines including IL-1*β* and tumor necrosis factor-*α* (TNF-*α*) have a significant impact on the NF-*κ*B signaling pathway by strongly inhibiting the expression of PI16. It is therefore likely that PI16 is an endogenous protective cardiokine, which could be applied as a therapeutic target in heart diseases, such as HF and hypertrophic cardiomyopathy.

### 2.10. Neurotrophins

Mesoscopic astrocyte-like neurotrophic factor (MANF), an endoplasmic reticulum (ER) stress protein secreted by cardiomyocytes, protects cells against stress in a paracrine/autocrine manner. ER stress could activate transcription factor 6 (ATF6), which induces the expression and secretion of MANF [72]. It has also been reported that MI activates MANF expression in cardiomyocytes and nonmyocytes, attenuating cardiac hypertrophy and myocardial ischemic injury. Additionally, a preclinical study showed that MANF knockout increases myocardial ischemia after ischemia reperfusion [72]. In contrast, increasing the level of MANF by administering recombinant MANF protein protects against heart injury in mice [73]. An in vitro study using cultured cardiomyocytes indicated that MANF could exert inhibitory effects on stress-induced hypertrophy [74]. It is therefore likely that MANF is a beneficial cardiokine that assists with recovery following cardiac diseases.

Similar to MANF, cerebral dopamine neurotrophic factor (CDNF) is an ER stress protein that can be induced by the activation of ER stress in cultured cardiomyocytes [75]. In addition, overexpression of CDNF improves cell viability and protects cardiomyocytes against ER stress-induced apoptosis.

Neuron-derived neurotrophic factor (NDNF) [76], a proangiogenic secreted protein with a fibronectin type III domain, improves poor myocardial remodeling after infarction and enhances cardiac cell survival and angiogenesis through a focal adhesion kinase/Akt-dependent pathway. Mice intramuscularly injected with adenovirus vectors expressing NDNF exhibit better left ventricular systolic and diastolic function after MI, as well as enhanced capillary formation and reduced posterior cardiomyocyte apoptosis and hypertrophy. Consequently, treatment of cultured cardiomyocytes using recombinant NDNF could reduce apoptosis under hypoxic conditions.

Brain-derived neurotrophic factor (BDNF) is widely expressed in many nonneural tissues such as vascular endothelial cells and myocardial cells, and it plays a role in regulating vascular repair and promoting wound healing [77]. It plays an important role in the improvement of myocardial microcirculation after myocardial damage [78]. Myocardial ischemia preconditioning increases the expression of BDNF mRNA and protein in cardiomyocytes, suggesting that BDNF exerts a protective action against myocardial IR by reducing apoptosis and enhancing antioxidant activity in the heart [79]. Tropomyosin receptor kinase B (TrkB), a functional receptor of BDNF, mediates downstream signaling through dimerization with BDNF and intracellular kinase-specific tyrosine phosphorylation, thereby enhancing cardiac microvascular endothelial cell proliferation and survival [80, 81]. It is therefore possible that BDNF is helpful during myocardial repair by promoting neovascularization.

## 3. The Detrimental Role of Cardiokines in CVD

### 3.1. Angiotensin-II

Angiotensin-II (Ang-II) is mainly synthesized and released by the renin-angiotensin-aldosterone system (RAAS) [82]. Interestingly, a study by Chen et al. demonstrated that Ang-II could also be produced by cardiomyocytes and fibroblasts in the heart, which elicits biological effects through paracrine or autocrine pathways [83]. CFs are the key cells initiating the formation of myocardial fibrosis. Zhang et al. demonstrated that Ang-II has the potential to abnormally increase the growth of CFs, resulting in myocardial fibrosis, through a transient receptor potential melastatin-7 channel-mediated (TRPM7, calcium channels) inward calcium current [84]. In addition, Ang-II promotes the expression of the Ets-1 gene in CFs, which is involved in tissue fibrosis remodeling, in a time and concentration dependent manner via the Ang-II 1 receptor (AT1R), c-Jun N-terminal kinase (JNK), or ERK signaling pathway [85]. Furthermore, pretreatments using losartan (an AT1R inhibitor), PD98059 (an ERK inhibitor), or SP600125 (a JNK inhibitor) facilitate the inhibition of cell proliferation and myocardial fibrosis by significantly downregulating profibrogenic factors such as connective tissue growth factor (CTGF) and plasminogen activator inhibitor-1 (PAI-1) [86]. Similarly, Ang-II leads to cardiac diastolic dysfunction by inducing myocardial fatty acid oxidation [87]. Given the critical role of Ang-II in CHF, it may aid accurate diagnosis and act as a predictor for clinical outcomes [88, 89]. Moreover, NT-proBNP is highly associated with altered levels of Ang-II. Together, this evidence suggests that combined measurements of NT-proBNP with Ang-II could effectively improve diagnostic accuracy for CHF [90].

### 3.2. Interleukin-1*β*, Interleukin-6, and Interleukin-18

In contrast to the role of IL-33, some interleukins have a detrimental effect in heart diseases. A preliminary study indicated that IL-1*β* might contribute to the onset of cardiomyocyte hypertrophy [91] and that sustained high levels of IL-1*β* not only cause cardiac pump impairment but also aggravate undesirable cardiac remodeling [92]. Additionally, IL-1*β* induces the expression of nitric oxide (NO) synthase and weakens the positive effects of *β*-adrenergic agonists on cardiomyocytes [93, 94]. Furthermore, the level of IL-6 in the blood is elevated in patients with MI, and sustained excess IL-6 production leads to cardiac damage through glycoprotein 130 (gp130) [95, 96]. Circulating levels of IL-6 are also closely related to the severity of left ventricular dysfunction and are an effective predictor for subsequent clinical complications [97]. Moreover, IL-18 is an independent risk factor in the formation and development of plaques in atherosclerosis by reducing the stability of atherosclerotic plaques and ECM degradation [98, 99].

### 3.3. Tumor Necrosis Factor-*α*

Tumor necrosis factor-*α* (TNF-*α*) is expressed by myocardial cells under stress and it is a harmful cardiokine involved in atherosclerosis [100]. TNF-*α* is upregulated during CHF and it contributes to impaired myocardial contractility, cardiomyocyte apoptosis, and myocardial remodeling. More importantly, serum levels of TNF-*α* are associated with CHF severity [101, 102].

There are several studies on the relationship between the expression of TNF-*α* and IL-1*β* with secondary ventricular arrhythmias in patients with acute coronary syndromes [103, 104], in which TNF-*α* and IL-1*β* are significantly upregulated and the levels increase with the deterioration of ventricular arrhythmia. Consequently, TNF-*α* and IL-1*β* are helpful in predicting the occurrence of secondary ventricular arrhythmia in patients with acute coronary syndrome and could be applied as useful biomarkers in estimating the severity of ventricular arrhythmia. Three possibilities underlie these pathological mechanisms: (1) TNF-*α* may be related to the opening of calcium ion channels in cardiomyocytes through a signal transduction pathway such as phospholipase A2/arachidonic acid (PLA2/AA), which affects cardiomyocyte repolarization and impairs contraction [105, 106]; (2) TNF-*α* could alter the potassium channels of cardiomyocytes via a protein kinase A (PKA) signaling pathway and inhibit rectifying potassium currents, ultimately causing myocardial abnormalities [107]; (3) TNF-*α* has also been shown to downregulate the expression of connexin 40 (Cx40) in gap junctions, thereby affecting intercellular communication and inducing arrhythmias [108].

### 3.4. Fibroblast Growth Factor 23

As a member of the FGF family, FGF23 derived from injured myocardial tissues, in contrast with the beneficial role of FGF21, promotes fibrosis and diastolic dysfunction after MI or IR [109]. In this pathological process, FGF23 is frequently accompanied by the activation of *β*-Klotho and TGF-*β* [110]. Recombinant FGF23 administration can directly induce pathological cardiac hypertrophy [111]. Furthermore, FGF23 elevation in the circulation is highly associated with an increased risk of cardiovascular events, such as myocardial ischemia, stroke, and cardiovascular disease-related deaths [112]. Intriguingly, the ERK1/2 pathway plays a critical role in FGF23 function and could enhance phosphate-mediated vascular calcification by promoting osteoblastic differentiation [113].

### 3.5. Matrix Metalloproteinases

Matrix metalloproteinases (MMPs) are a group of proteins that are capable of selectively degrading ECM and regulate most of the ECM remodeling in CHF patients via cardiac remodeling and left ventricular dilatation [114]. All MMPs are negatively regulated by tissue inhibitors of metalloproteinase (TIMPs), and MMP/TIMP imbalance may result in heart disease [115].

MMPs are dramatically increased during HF progress and recovery [116]. In sheep models simulating the process of left ventricular hypertrophy, failure, and recovery, different MMP subtypes and their TIMP inhibitors were abnormally regulated during the process of myocardial ECM remodeling, thereby affecting the development of HF and ventricular remodeling [117]. In addition, the levels of MMP-2 and MMP-9 in patients with coronary atherosclerotic heart disease are significantly increased, while exogenous inhibitors restrain the expression and activity of MMPs to maintain the stability of atherosclerotic platelets [117]. Together, this evidence indicates that MMPs are harmful cardiokines, which exacerbate the prognosis of heart disease. TIMPs may act as new therapeutic targets for cardiac diseases via inhibition of MMPs, but this approach requires further investigation.

### 3.6. Platelet-Derived Growth Factors

Platelet-derived growth factors (PDGFs) are commonly expressed in the myocardium and interstitial fibroblasts [118]. PDGFs stimulate pathological hyperplasia of fibroblasts and convert them into myofibroblasts by activating specific receptors (PDGFR-*α* and PDGFR-*β*), resulting in the production of a significant amount of collagen, which is involved in the development of fibrosis [119, 120]. PDGF is also closely related to the occurrence and progression of coronary atherosclerosis, in which PDGF-D affects the stability of coronary atherosclerotic plaques [121]. A previous study indicated that PDGF could promote the accumulation of smooth muscle cells and the formation of foam cells in atherosclerosis [122]. In addition, PDGF has the potential to induce the division and proliferation of damaged epithelial or endothelial cells, resulting in the subsequent aggravation of atherosclerosis [123].

## 4. Undetermined Role of Cardiokines in CVD

### 4.1. Transforming Growth Factor-*β*

Transforming growth factor-*β* (TGF-*β*) is secreted by multiple cell types including cardiomyocytes, which affects the regulation of cell growth and differentiation. It has been reported that TGF-*β* is a regulator of neutrophil infiltration and can clean up the necrotic cells and matrix caused by the inflammatory response during MI recovery. TGF-*β* is therefore significantly associated with undesirable ventricular remodeling after infarction [124–126]. Recently, accumulating evidence has shown that members of the TGF-*β* superfamily including growth differentiation factor-15 (GDF-15), myostatin, and activin A play a cardioprotective role in maintaining normal cardiac homeostasis and inhibiting myocardial hypertrophy [34, 127–129]. In addition, FSTL1 (mentioned earlier in the article) is a regulatory factor for GDF-15 in cultured cardiac myocytes or mouse hearts, where it protects against cardiac stress [130], while overexpression of FSTL3 can reverse the effects of activin A to promote cardiomyocyte survival and inhibit cardiomyocyte hypertrophy [131, 132], suggesting that activin A and FSTL3 exert counteractive effects in regulating myocardial cell growth and fibrosis and the response to ischemic stress. Collectively, these findings deepen our understanding of cardiokine networks.

In contrast, several studies have demonstrated that TGF-*β* inhibition in the late stage of MI may improve myocardial remodeling, while TGF-*β* inhibition in the early stage has opposite effects [133, 134], suggesting that the activation of TGF-*β* may protect the heart from ischemic injury at an early stage, but its beneficial effect is later diminished. Similarly, Rainer et al., using a mouse model of selective TGF-*β* receptor 1 or 2 knockout, demonstrated that the inhibitory effects of TGF-*β* signaling on cardiomyocytes could significantly suppress neutrophil aggregation in the heart, restrain the inflammatory reaction, prevent cardiac rupture after MI, and improve remodeling [126]. Selective TGF-*β* inhibition improves ventricular remodeling by directly reducing the production of proinflammatory cytokines/chemokines and inhibiting neutrophil activation and migration through inducing the synthesis of other protective cardiokines. However, specific and pleiotropic characteristics of TGF-*β* may contribute to various potential side-effects of nonselective inhibition [135]. The adverse roles of TGF-*β* at different stages of MI could also increase its complexity for clinic application, so careful assessments must be undertaken to determine how TGF-*β* could be adopted in clinical treatment to improve ventricular remodeling.

### 4.2. C1q/TNF-Related Protein 9

C1q/TNF-related protein 9 (CTRP9) is a novel cardiokine with high relative homology to adiponectin (APN) [136], which is primarily secreted by adipose tissue and cardiac endothelial cells [137]. CTRP9 can maintain homeostasis and improve the prognosis of heart disease via its inhibitory effects on inflammation, post-IR injury, and ventricular remodeling after MI [138–141]. Kambara et al. [138, 142] demonstrated that CTRP9 levels are reduced by nearly 50% in mice following myocardial IR injury with an increase of blood levels of free fatty acid (FFA). An exogenous injection of CTRP9 could dramatically attenuate MI and improve cardiomyocyte apoptosis after myocardial ischemia, suggesting that CTRP9 is a protective cardiokine in the cardiovascular system. In contrast, it was also found that heterozygous or homozygous CTRP9 knockout mice showed less cardiac hypertrophy and pulmonary congestion after pressure overload, as well as better systolic cardiac function, compared with wild-type mice [137]. Moreover, CTRP9 overexpression induces serious cardiac dysfunction, suggesting that CTRP9 might exert a deleterious effect on cardiac hypertrophy and HF. Since the role of CTRP9 in cardiac diseases has not been fully determined, future studies on clarifying how CTRP9 triggers different signaling pathways during the pathological process of cardiac hypertrophy are required.

## 5. Conclusions

The physiological role of cardiokines has been attracting more attention since cardiokines have shown significant potential as biomarkers to evaluate cardiac function and as therapeutic targets for cardiac diseases. It has been suggested not only that cardiokines have physiological effects on cardiac tissues, but that they may also exert regulatory effects on peripheral organs and tissues [143, 144]. Further detailed studies on the role of cardiokines in the crosstalk between the heart and peripheral organs are required. In addition, the regulatory effects of cardiokines are often complex, as they can exert bidirectional actions to promote the repair of cardiac injury and/or aggravate an imbalance of cardiac function. Since the physiological role of cardiokines in cardiac diseases is not fully determined, additional studies are warranted.

## Figures and Tables

**Table 1:** Summary of the physiological roles of cardiokines in cardiac diseases.

Cardiokine	Beneficial or detrimental	Action mechanisms	Dose- response	Types of cardiac diseases	Predictor
Natriuretic peptide					
ANP [11]	Beneficial	-	Yes	HF	-
BNP [3, 12–15]	Beneficial	-	Yes	HF	Yes
Interleukin					
IL-33 [23–27]	Beneficial	IL-33/ST2, sST2	-	HF, MI, CH, MF	Yes (ST2)
IL-6 [95–97]	Detrimental	gp130	Yes	MI	Yes
IL-18 [98, 99]	Detrimental	-	-	CAHD	-
IL-1*β* [91–94]	Detrimental	NO synthase	-	CH, ACS	-
Follistatin					
FSTL1 [30–38]	Beneficial	AMPK, BMP-4	-	MI, CH, HF	-
FSTL3 [131, 132]	Detrimental	-	-	CH	-
FGF					
FGF21 [39–45]	Beneficial	*β*-Klotho, ERK	-	CAHD	-
FGF23 [109–113]	Harmful	ERK1/2	-	MF, CH	-
Sfrp					
Sfrp-3 [46–50]	Beneficial	Wnt signaling	Yes	HF, MI	-
Sfrp-2 [51–54]	Protective	Wnt signaling, BMP-1/Tolloid-like metalloproteinase	-	MI	-
MIF [55–59]	Beneficial (the cardiogenic)	-	-	MI	-
NRG [60–62]	Beneficial	-	-	MI, MF	-
ADM [63–69]	Beneficial	-	Yes	MI, CAHD, MI	Yes
PI16 [70, 71]	Beneficial	KLF2, ERK5	-	CH, HF	-
Neurotrophins					
MANF [72–74]	Beneficial	ATF6	-	MI, CH	-
CDNF [75]	Beneficial	-	-	MI	-
NDNF [76]	Beneficial	Focal adhesion kinase/Akt	-	MI	-
BDNF [77–81]	Beneficial	TrkB	-	MI	-
Ang-II [82–86]	Detrimental	TRPM7, AT1R, JNK,ERK	-	MF, HF	-
TNF-*α* [100–108]	Detrimental	PLA2/AA, PKA, Cx40	Yes	HF, ACS, Arrhythmia	-
MMPs [114–117]	Detrimental	TIMPs	-	HF, CAHD, ACS	-
PDGF [118–123]	Detrimental	PDGFR-*α* and PDGFR-*β*	-	MF, CAHD	-
TGF-*β* [34, 124–135]	Undetermined	TGF-*β* receptor 1/2	-	MI	-
CTRP9 [135–142]	Undetermined	gCTRP9, AdipoR1, AMPK, Akt	-	MI, HF, CH	-

ACS: acute coronary syndrome; ADM: adrenomedullin; Ang-II: angiotensin-II; AMPK: adenosine 5′-monophosphate-activated protein kinase; ANP: atrial natriuretic peptide; AT1R: Ang-II 1 receptor; ATF6: activating transcription factor 6; BDNF: brain-derived neurotrophic factor; Bmp1: bone morphogenic protein 1; BNP: brain natriuretic peptide; CAHD: coronary atherosclerotic heart disease; CDNF: cerebral dopamine neurotrophic factor; CH: cardiac hypertrophy; CTRP9: C1q/TNF-related protein 9; Cx40: connexin 40; ERK: extracellular regulated protein kinases; FGF: fibroblast growth factor; FSTL1: follistatin-like 1; GDF-15: growth differentiation factor-15; gp130: glycoprotein 130; HF: heart failure; IL: interleukin; JNK: c-Jun N-terminal kinase; MANF: mesoscopic astrocyte-like neurotrophic factor; MF: myocardial fibrosis; MI: myocardial infarction; MIF: macrophage migration inhibitory factor; MMPs: matrix metalloproteinases; NDNF: neuron-derived neurotrophic factor; NO: nitric oxide; PDGF: platelet-derived growth factor; PI16: protease inhibitor 16; PKA: protein kinase A; PLA2/AA: phospholipase A2/arachidonic acid; Sfrp-3: secreted frizzled-related protein-3; TGF-*β*: transforming growth factor-*β*; TIMP: tissue inhibitor of metalloproteinase; TNF-*α*: tumor necrosis factor-*α*; TRPM7: transient receptor potential melastatin-7 channels; TSC-36: transforming growth factor *β*-stimulated clone 36.

## Abbreviations

ACS: Acute coronary syndrome
ADM: Adrenomedullin
Ang-II: Angiotensin-II
AMPK: Adenosine 5′-monophosphate-activated protein kinase
ANP: Atrial natriuretic peptide
APN: Adiponectin
AT1R: Ang-II 1 receptor
ATF6: Activating transcription factor 6
BDNF: Brain-derived neurotrophic factor
Bmp1: Bone morphogenic protein 1
BNP: Brain natriuretic peptide
CAHD: Coronary atherosclerotic heart disease
CDNF: Cerebral dopamine neurotrophic factor
CFs: Cardiac fibroblasts
CH: Cardiac hypertrophy
CHF: Congestive heart failure
CMEC: Cardiac microvascular endothelial cells
CTGF: Connective tissue growth factor
CTRP9: C1q/TNF-related protein 9
CVD: Cardiovascular disease
Cx40: Connexin 40
ECM: Extracellular matrix
EF: Ejection fraction
EGF: Epidermal growth factor
ER: Endoplasmic reticulum
ERK: Extracellular regulated protein kinases
FFA: Free fatty acid
FGF: Fibroblast growth factor
FSTL1: Follistatin-like 1
GDF-15: Growth differentiation factor-15
gp130: Glycoprotein 130
HF: Heart failure
IL: Interleukin
IR: Ischemia/reperfusion
JNK: c-Jun N-terminal kinase
MANF: Mesoscopic astrocyte-like neurotrophic factor
MF: Myocardial fibrosis
MI: Myocardial infarction
MIF: Macrophage migration inhibitory factor
MMPs: Matrix metalloproteinases
NDNF: Neuron-derived neurotrophic factor
NO: Nitric oxide
NRG: Neuregulin
NYHA: New York Heart Association
PAI-1: Plasminogen activator inhibitor-1
PDGF: Platelet-derived growth factor
PI16: Protease inhibitor 16
PKA: Protein kinase A
PLA2/AA: Phospholipase A2/arachidonic acid
RAAS: Renin-angiotensin-aldosterone system
Sfrp-3: Secreted frizzled-related protein-3
TGF-*β*: Transforming growth factor-*β*
TIMP: Tissue inhibitor of metalloproteinase
TNF-*α*: Tumor necrosis factor-*α*
TRPM7: Transient receptor potential melastatin-7 channels
TSC-36: Transforming growth factor *β*-stimulated clone 36.
